# Regression toward the mean – a detection method for unknown population mean based on Mee and Chua's algorithm

**DOI:** 10.1186/1471-2288-8-52

**Published:** 2008-08-07

**Authors:** Thomas Ostermann, Stefan N Willich, Rainer Lüdtke

**Affiliations:** 1 Department of Medical Theory and Complementary Medicine, University of Witten/Herdecke, Gerhard-Kienle-Weg 4, 58313 Herdecke, Germany; 2 Institute of Social Medicine, Epidemiology, and Health Economics, Charité University Medical Center, Berlin, Germany; 3 Karl and Veronica Carstens Foundation, Am Deimelsberg 36, 45276 Essen, Germany

## Abstract

**Background:**

Regression to the mean (RTM) occurs in situations of repeated measurements when extreme values are followed by measurements in the same subjects that are closer to the mean of the basic population. In uncontrolled studies such changes are likely to be interpreted as a real treatment effect.

**Methods:**

Several statistical approaches have been developed to analyse such situations, including the algorithm of Mee and Chua which assumes a known population mean *μ*. We extend this approach to a situation where *μ *is unknown and suggest to vary it systematically over a range of reasonable values. Using differential calculus we provide formulas to estimate the range of *μ *where treatment effects are likely to occur when RTM is present.

**Results:**

We successfully applied our method to three real world examples denoting situations when (a) no treatment effect can be confirmed regardless which *μ *is true, (b) when a treatment effect must be assumed independent from the true *μ *and (c) in the appraisal of results of uncontrolled studies.

**Conclusion:**

Our method can be used to separate the wheat from the chaff in situations, when one has to interpret the results of uncontrolled studies. In meta-analysis, health-technology reports or systematic reviews this approach may be helpful to clarify the evidence given from uncontrolled observational studies.

## Background

Regression to the mean (RTM) first described by Galton [1] is a statistical phenomenon broadly discussed when it comes to measure changes in the course of time. It occurs in situations of repeated measurements when extremely large or small values are followed by measurements in the same subjects that on average are closer to the mean of the basic population. Such changes are likely to be interpreted as a real drift, although they just might be artificial coming from the fact that the sampling of values was not random but selected.

RTM affects all fields of life science, when effects of an intervention have to be evaluated in an uncontrolled longitudinal setting. Medical rehabilitation programmes for example, often are evaluated for their ability to restore the patient's ability to work. Unaware of RTM effects a patient's recovery typically is interpreted as a treatment effect [2]. Other examples include the evaluation of asthma disease management programmes [3] or cholesterol screening [4].

The discussion about the development of methods to detect RTM in observational studies is still vital [5]. This is especially true for the results of complementary therapies, which are often claimed to be a mixture of RTM effects, non-specific (placebo) effects, the effects of a previous or concomitant conventional treatment and the actual effectiveness of the complementary treatment [6,7].

In the last two decades several methods for detecting RTM have been developed both for the case of normal distributed data [8,9] as well as for the non-parametric case [10,11]. Most of these methods deal with common situations of truncated sampling, i.e. only those members which have a first measurement beyond (or below) a predefined cut-point are sampled. The approach we focus on in this paper is a straightforward method developed by Mee and Chua [12] based on classical t-test statistics and a linear regression model. This method does not depend on truncated sampling but requires the knowledge of the true mean *μ *in the target population. If *μ *can be obtained, this approach has already been proven to distinguish between RTM-effects and treatment effects in clinical study reality [13,14]. However, the basic necessity of a population mean is quite obstructive and often such a value can not be determined. In this paper, we therefore revisit the approach of Mee and Chua and extend it to a situation where no population mean is available but evidence for or against a treatment effect is needed when RTM is present.

## Notations

In the following we consider two measurements of one quality (e.g. physiological parameters like blood pressure, or quality of life scores): The random variables Y_1 _(Y_2_) denote these values/scores before (after) an intervention. Realisations of Y_1 _and Y_2 _are denoted as y_1 _and y_2_. We assume that both measurements follow a bivariate normal distribution with means *μ*_1 _and *μ*_2_, a common variance *σ*_1_^2 ^= *σ*_2_^2 ^= *σ*^2 ^and a correlation *ρ*. In the case of no change in distributions between the two repeated measurements (i.e. there is a common mean *μ *= *μ*_1 _= *μ*_2 _for Y_1 _and Y_2_) the conditional expectation of Y_2_, given Y_1 _= y_1_, can be easily calculated as

(1) *E*(*Y*_2_|*Y*_1 _= *Y*_1_) = *μ *+ *ρ*(*Y*_1 _- *μ*)

Equation (1) describes the RTM effect mathematically: if Y_1 _is large (Y_1 _> *μ*) then Y_2 _is expected to be smaller (if only 0 ≤ *ρ *< 1), and if Y_1 _is smaller than the mean then Y_2 _is expected to be larger. In both cases Y_2 _is expected closer than Y_1 _to the mean.

Mee and Chua exploited equation (1) to construct a test which allows to differentiate between the RTM effect and an intervention effect *τ*. In detail, they rewrote (1) as a regression equation and introduced *τ *as acting additively to the RTM effect:

(2) *Y*_2 _= *μ *+ *τ *+ *ρ*(*Y*_1 _- *μ*) + *ε*

where *ε *is a normally distributed random error.

Note that equation (2) extends the original model: *ρ *now denotes not a correlation but is interpreted as a slope where |*ρ*| > 1 is allowed.

Mee and Chua's test involves regressing the outcome values Y_2 _after therapy on X = Y_1_-*μ*, where *μ *is assumed to be fixed and known. By applying simple linear regression techniques the intercept *β*_0 _= *μ*+*τ *and the slope *ρ *are estimated. Subsequently, using t-test statistics the hypothesis is tested that the intervention has an additive benefit, i.e. H_0_: *β*_0 _= *μ *is tested against H_1_: *β*_0 _= *μ *+ *τ *with *τ *≠ 0. Using Mee and Chuas notations the single steps of their algorithm are as follows:

1. Calculate X = Y_1_-*μ*

2. Estimate the parameters *β*_0 _and *ρ *from the linear regression model of Y_2 _on X

3. Estimate the treatment effect $\hat{\tau }$ by subtracting *μ *from ${\hat{\beta }}_{0}$, the estimate of *β*_0_

4. Calculate the test-statistic

(3) t=t(μ)=(β^0−μ)s2(1n+X¯2/∑i=1n(Xi−X¯)2)

where s^2 ^is the mean squared error in the simple regression analysis of variance, X_i _denotes the value of X in the i-th patient, i = 1,..., n, and $\bar{X}$ is the mean of all X_i_.

5. Compare t with the appropriate t-distribution with (n-2) degrees of freedom to obtain a p-value p = p(*μ*).

This procedure is equivalent to a linear regression analysis of Y_2_-*μ *on Y_1_-*μ*. In this model equation (3) describes the test whether the intercept differs from null (H_0_: *τ *= 0), which can be carried out by most statistical standard software.

The calculations of the test statistic t(*μ*) may be even more simple if one rewrites equation (3) in terms of simple statistics, such as the sample means ${\bar{Y}}_{1}$ and ${\bar{Y}}_{2}$, the sample variances $s_{Y_{1}}^{2}$ and $s_{Y_{2}}^{2}$, the correlation $r_{Y_{1}Y_{2}}$ of Y_1 _and Y_2_, or their respective covariance $s_{Y_{1}Y_{2}}$.

(4) $$t(\mu )=\sqrt{n(n-2)}\frac{s_{Y_{1}}^{2}{\bar{Y}}_{2}-s_{Y_{1}Y_{2}}{\bar{Y}}_{1}+(s_{Y_{1}Y_{2}}-s_{Y_{1}}^{2})\mu }{\sqrt{\left(s_{Y_{1}}^{2}s_{Y_{2}}^{2}-s_{Y_{1}Y_{2}}^{2}\right)\left((n-1)s_{Y_{1}}^{2}+n{\left({\bar{Y}}_{1}-\mu \right)}^{2}\right)}}$$

## A simple extension of Mee and Chua's test

Mee and Chua's test can be extended to overcome the limitation that the population mean *μ *must be assumed to be known. In the case of unknown *μ*, we suggest to vary *μ *systematically over a range of reasonable values and to perform the above described procedure for each *μ *separately. Afterwards, defined statistics, such as t(*μ*), p(*μ*) or the estimated treatment effect $\hat{\tau }$(*μ*), can be plotted against *μ *which should give an overall impression how RTM affects the data.

The graph of t(*μ*) as defined in equation (4) can be analysed in some more detail. First, after standard calculations it can be seen that t(*μ*) converges to a fixed value when *μ *approaches infinity:

(5) t(μ)→μ→±∞∓n−2sY1Y2−sY12sY12sY22−sY1Y22=±n−2(sY1sY2−rY1Y2)1−rY1Y22

Moreover, assuming that ${\bar{Y}}_{1}$ ≠ ${\bar{Y}}_{2}$, differentiation with respect to *μ *shows that t(*μ*) has only one extremum *t*_*ext *_= t(*μ*_*ext*_) which can be found at

(6) $$\mu_{ext}={\bar{Y}}_{1}+\frac{n-1}{n}\frac{\left(r_{Y_{1}Y_{2}}\sqrt{s_{Y_{2}}^{2}s_{Y_{1}}^{2}}-s_{Y_{1}}^{2}\right)}{\left({\bar{Y}}_{2}-{\bar{Y}}_{1}\right)}$$

If ${\bar{Y}}_{1}$ = ${\bar{Y}}_{2}$, t(*μ*) is strictly monotone and no extremum can be found at all. If ${\bar{Y}}_{1}$ <${\bar{Y}}_{2}$ substituting *μ*_*ext *_into equation (4) yields equation (7) which can be shown to define a maximum *t*_*max*_:

(7) $$t_{\max}=t(\mu_{ext})=\sqrt{\frac{\left(n-2\right)}{s_{Y_{2}}^{2}(1-r_{Y_{1}Y_{2}}^{2})}}\frac{n{\left({\bar{Y}}_{2}-{\bar{Y}}_{1}\right)}^{2}+(n-1){\left(r_{Y_{1}Y_{2}}s_{Y_{2}}-s_{Y_{1}}\right)}^{2}}{\sqrt{n(n-1){\left({\bar{Y}}_{2}-{\bar{Y}}_{1}\right)}^{2}+{\left(n-1\right)}^{2}{\left(r_{Y_{1}Y_{2}}s_{Y_{2}}-s_{Y_{1}}\right)}^{2}}}$$

If ${\bar{Y}}_{1}$ > ${\bar{Y}}_{2}$*μ*_*ext *_defines a minimum with t_min _= -t_max _in (7).

For large n equations (6) and (7) simplify into

$$\begin{array}{ccc} \mu_{ext}={\bar{Y}}_{1}+\frac{\left(r_{Y_{1}Y_{2}}\sqrt{s_{Y_{2}}^{2}s_{Y_{1}}^{2}}-s_{Y_{1}}^{2}\right)}{\left({\bar{Y}}_{2}-{\bar{Y}}_{1}\right)} & \text{and} & t_{ext}=\sqrt{\frac{n\left({\left({\bar{Y}}_{2}-{\bar{Y}}_{1}\right)}^{2}+{\left(r_{Y_{1}Y_{2}}s_{Y_{2}}-s_{Y_{1}}\right)}^{2}\right)}{(1-r_{Y_{1}Y_{2}}^{2})s_{Y_{2}}^{2}}} \end{array}$$

In most situations it will turn out that p(*μ*_*ext*_) falls below the predefined significance level *α*. Then immediately the question arises for which *μ*'s this is also true, i.e. for which region of *μ *a significant treatment effect can be expected. Setting t(μ*) = t_n-2;1-*α*/2 _(the 1-*α*/2-quantile of a t-distribution with n-2 degrees of freedom) this leads to a quadratic equation in μ* which can be solved by conventional techniques yielding solutions $\mu_{1}^{*}$ and $\mu_{2}^{*}$. As these formulas are somewhat lengthy we refrain from reporting them here.

For the following assume that there exist solutions $\mu_{1}^{*}$ and $\mu_{2}^{*}$, i.e. there is at least one *μ *which yields to a significant treatment effect. In this case it can be seen from the formulas mentioned above that each *μ *outside the interval [$\mu_{1}^{*}$; $\mu_{2}^{*}$] leads to a significant treatment effect, if and only if

(8) n−2(sY1sY2−rY1Y2)1−rY1Y22>tn−2;1−α/2

This is usually true for large n. If n is small equation (8) holds if $r_{Y_{1}Y_{2}}$ is small, or $s_{Y_{1}}^{2}$ is considerably larger than $s_{Y_{2}}^{2}$. Otherwise, all *μ *inside this interval lead to a significant treatment effect if and only if equation (8) does not hold.

All equations presented only depend on the number of subjects *n *and simple sample statistics. It is therefore easy to encode them in standard software programs which we have done for MS-EXCEL ^® ^and SAS ^®^. The implementation in SAS is solved as a macro (see Additional file 1). It is meant for situations when individualised data is available. The EXCEL solution should be considered when the sample statistics can be drawn from the paper but individual data is not available. Both programs are appended to this manuscript.

## Examples

We apply the method developed above to three examples. First, we look for the data given in the original work of Mee and Chuas classical approach:

### Example 1

Table 1 provides the individual data originally taken from McClave and Dietrich [15]. It comprises the scores of n = 8 students who failed to pass a test to receive their high school diploma. These students were encouraged to visit a refresher course and to retake an equivalent test afterwards. As the mean (± standard deviation) test score increases from 57.4 ± 7.0 to 60.4 ± 8.1 points one might conclude that the refresher course is effective, a point of view which is supported by a paired t-test which results in a one-sided p-value of 0.0428.

**Table 1:** Data of a repeated language-test after a special training (Example 1).

Student	Before	After
1	45	49
2	52	50
3	63	70
4	68	71
5	57	53
6	55	61
7	60	62
8	59	67

On the other side, the analysed data was not drawn from the whole population but only from the lower extremes of the distribution (the students who performed worst). Thus RTM is likely to occur and should be addressed in a formal analysis. In their paper, Mee and Chua assumed a true mean of *μ = 75 *and calculated from equation (3) a value of *t = t(75) = 1.08*, which gives a one-sided p-value of p = p(75) = *P(t_6 _> 1.08) = 0.16*. They concluded that the observed changes might be attributed to RTM and an intervention effect could not be confirmed.

**Figure 1:**
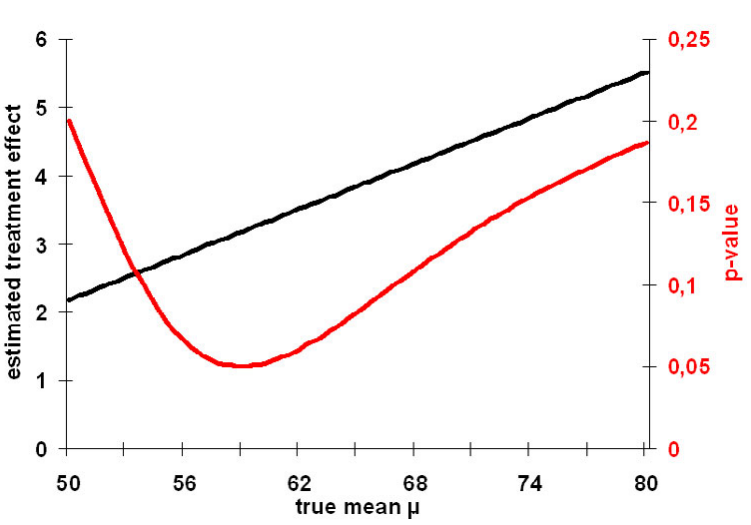
Graphs for *p(μ) and *$\hat{\tau }$(*μ*) based on example 1 given in table 1.

Following the approach we suggested here, one might wonder whether this result is sensitive to the assumption of *μ *= *75*. In other words one should calculate if there would have been a chance of an intervention effect if another *μ *had been chosen. Fig. 1 shows the values for p(*μ*) based on the data given in table 1 within a range from 30 <*μ *< 80. From equation (4) and (5) the maximum value for t is given at *μ*_max _= 58.96, with a t-value of t_max _= 1.938. This finally leads to a corresponding one sided p-value of p_min _= p(*μ*_max_) = 0.0504. Hence, we can surprisingly conclude, that independent of any given *μ *no intervention-effect can be confirmed in this group of students. Thus, the data does not support the hypothesis, that the special course to refresh the language skills is not suitable for the given student profile that failed in the first exam.

### Example 2

The next example deals with homeopathy, one of the most frequently used and controversial systems of complementary and alternative medicine. Homeopathy is based on the 'principle of similars', whereby highly diluted preparations of substances that cause symptoms in healthy individuals are used to stimulate healing processes in patients who have similar symptoms when ill.

Recently, Witt et al. [16] presented an uncontrolled cohort study which found marked beneficial health effects in nearly 3.000 chronic diseased adults when homeopathically treated. Of those, 214 patients suffered from migraine. Within two years their quality of life, as measured by the SF-36 physical summary score, increased from 44.3 ± 11.8 to 49.4 ± 12.3 score points. The question arises whether this increase is due to RTM or can be attributed to a true intervention effect.

**Figure 2:**
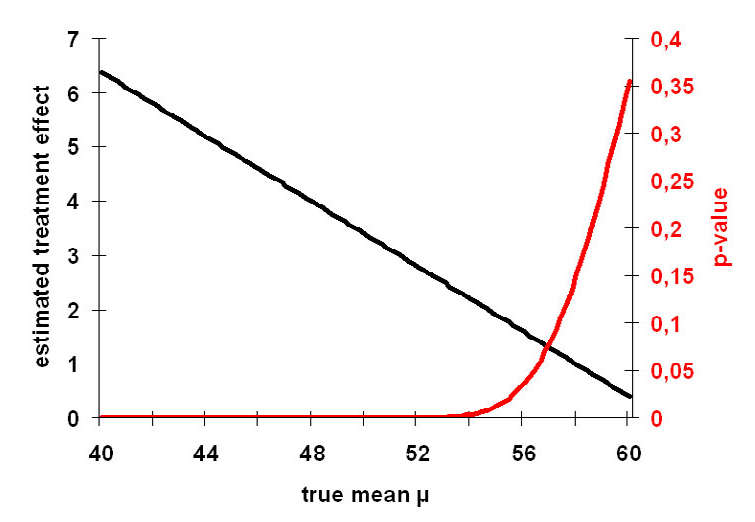
Graphs for *p(μ) and *$\hat{\tau }$(*μ*) based on example 2 (Becker-Witt [16]).

Fig. 2 shows that the p-values drawn from the Mee-Chua-test are far below 0.025 when the true mean is below 55 score points. Thus, in these situations a significant intervention effect can be confirmed. Having in mind that the true (healthy) population in Germany has a mean SF-36 physical summary score of 50.24 [17] it seems very unlikely that the true mean in our (diseased) target population is bigger than 55 points. Consequently, our analyses show unambiguously, that the observed effect in this study cannot only attributed to RTM.

### Example 3

Our method can be extended for seperating the wheat from the chaff in situations, when one has to interpret the results of uncontrolled studies. For example, one might think of a simple voting when classifying the possibility of a treatment effect in "never" "unlikely", "probably" and "most likely". Especially in meta-analysis, health-technology reports or systematic reviews, this approach can be quite helpful to clarify the evidence given from observational studies. This can be demonstrated in three uncontrolled studies on Bosentan treatment for patients with pulmonary arterial hypertension (PAH). The main outcome parameter in PAH-studies is usually given by the 6-minute walk distance (6MWD) which in our chosen studies was measured at baseline and after a treatment period of 16 weeks. As the correlation between the repeated measurements was not given, we ran our algorithm with three levels of correlations: high (r = 0.8), moderate (r = 0.5), and low (r = 0.2) correlation. Table 2 provides the regions of significance which are based on the intervals [$\mu_{1}^{*}$; $\mu_{2}^{*}$].

**Table 2:** Regions of significance and voting for a positive treatment effect in three uncontrolled studies on Bosentan in PAH based on the approach presented in this paper

Study	N	6MWD at Baseline	6MWD at week 16	correlation	region of significance	Vote of significance
Souza et al. 2005 [18]	15	396 ± 135	434 ± 137	r = 0.2	[0;337] & [672;∞]	Unlikely
				r = 0.5	[0;300]	Unlikely
				r = 0.8	-	Never

Provencher et al. 2006 [19]	99	322 ± 105	364 ± 109	r = 0.2	[0;347] & [410;∞]	Probably
				r = 0.5	[0;367] & [481;∞]	Probably
				r = 0.8	[0;448] & [1292;∞]	Most likely

Apostolopoulou et al. 2006 [20]	21	416 ± 105	459 ± 101	r = 0.2	[0;412] & [575;∞]	Unlikely
				r = 0.5	[0;420] & [820;∞]	Unlikely
				r = 0.8	[0;466]	Probably

(N- number of patients, 6MWT-6-minute walk distance, data before and after intervention is mean ± standard deviation, region of significance: only 6MWD values > 0 reported)

In most cases the region of significance is split into two parts: The upper part (*μ *is large) describes the region where a huge RTM effect is expected, larger than the actual difference of means, and a negative treatment effect (*τ *< 0) can be confirmed. For example, assuming a correlation of r = 0.5 in Provencher's trial the region of significance includes all values above 481 meters, saying that Bosentan has a significantly (p < 0.05) negative effect on the patient's 6MWD if only the true mean 6MWD is above this value in the population of interest. This part of the region is of no further interest in our example, because here we are only interested in the one-sided hypothesis whether Bosentan can increase the patient's 6MWD. In other situations however a two-sided hypothesis might be more appropriate.

The lower part of the region of significance includes values of *μ *where a positive treatment effect (*τ *> 0) can be confirmed. This is usually true when *μ *is considerably smaller than the baseline mean and the RTM effect pulls the values into the wrong direction. Again this region is of no further interest, because it describes a unrealistic situation. For example, in Provencher's trial the region of significance includes all values below 367 meters (assuming r = 0.5), saying that Bosentan does significantly (p < 0.05) incrase the patient's 6MWD if only the true mean 6MWD is below this value in the population of interest. But, values of 100 or 200 meters are exeptionally small, it is therefore unrealistic to assume that the mean 6MWD lies in this region.

What is left, is that part of the region of significance where a positive treatment effect can be confirmed for values of *μ *which are larger than the 6MWD mean at baseline. This usually occurs when the correlation is high, RTM effects are expected to be relatively small and the actual group changes can be predominantly attributed to the treatment effect. This is true in Provencher's trial (assuming r = 0.5), where the lower part of the region of significance exceeds 322 metres, the mean baseline value in the study population.

Having this in mind, we voted a treatment effect to be "unlikely" in the study of Souza et al [18], because Mee and Chua's modified t-test fails to reach a level of significance in realistic situations. In contrast, in both other studies a treatment effect of Bosentan is probable [19] or even most likely [20], at least when correlation is high (i.e. r = 0.8).

Interestingly, in all three studies the phenomenon described in equation (8) can be studied: Whenever the correlation approaches 1 the region of significance changes from a bipartite region to an interval [$\mu_{1}^{*}$; $\mu_{2}^{*}$] where treatment effects can be confirmed for values within this interval but not outside. An intuitive explanation for this phenomenon may be the following:

a) If *μ *is very small and the correlation r increases then the RTM effect decreases and finally is not far below the actual group difference. The estimated treatment effect is still positive but now cannot be confirmed statistically.

b) If *μ *is very large similar arguments hold. Again the RTM effect decreases when the correlation r increases and finally is roughly in the same range as the actual group difference. Consequently, a statistical confirmation of a treatment effect (whose estimate is still negative) fails.

c) If *μ *lies within the range of the baseline and the follow up mean, the RTM effect is small, but very similar to the acutal group change. If r increases the RTM effect becomes even smaller and neglectable, such that all actual group change can be interpreted as a treatment effect.

## Discussion

In this paper, we have developed a straight-forward method based on Mee and Chuas modified t-test to detect, whether a change in a uncontrolled repeated measurement-situation after an intervention in a selected population is due to RTM or to a specific treatment effect.

RTM is a statistical phenomenom often ignored, misunderstood or insufficiently appreciated and thus one of the the most fundamental sources of error in human reasoning in almost all scientific disciplines [21].

Since its first description from Galton in 1886 [1] RTM has been discussed by a variety of authors (a historical outline is given by Stigler [22]). Thorndike [23] to our knowledge was the first who developed mathematical formulas this problem based on a known population mean and normally distributed data. Almost at the same time Kelley [24] gave a theoretical framework known in classical test theory as Kelley's equation (see [21] for a deduction of this equation). Cohen [25] was the first who described the selection process in more detail. He distinguished between four kinds of sample in connection with bivariate nomal distributions: truncated, censored, selected, and complete samples. Based on his work Senn and Brown [26] derived maximum likelihood equations to estimate the RTM and the treatment effect. Das and Mulder [27] first left the assumption that the true underlying random variable Y_1 _is normally distributed and considered arbitrary (usually unimodal) continuous distributions. Their work still relied on the assumption of normally distributed measurement errors, which was renounced by Müller et al [11].

Unlike all of the above mentioned approaches our method does not need any information about the selection process. It therefore can also be used, if only the results of an intervention process are given, which unfortunately quite often occurs in papers presenting uncontrolled observational studies.

In contrast, when the selection process can be specified Mee and Chuas modified t-test (and hence our extension) generally has a low power, especially whenever all values of Y_1 _in the sample are in one extreme [12]. Assuming truncated sampling George et al. [28] contrasted the performance of the modified t-test with likelihood based alternatives. In their simulation studies the likelihood ratio-test appeared to be more powerful than the score test or the modified t-test.

The statistical model we propose here is based on the assumption that the population is in a steady state where the variance does not change in time and the correlation *ρ *is constant over the whole range of values. These are usual assumptions made in the literature on RTM which seem to be realistic in medical applications when the time between both observations is relatively small (see e.g. [26,29]). This has been doubted by Ragosa [30] who pointed out that the assumption of equal variances is essential in the discussion of RTM. If it does not hold and the variances increase over time then the conditional expectation of Y_2_, given Y_1 _= y_1_, is farther away from *μ *than Y_1_, so that regression indeed is "from the mean" not "to the mean" Ragosa thus called RTM a myth based on a mathematical tautology without any meaning in practice. In our examples, however, we found no hints, that the assumption of constant variances might be violated, the respective empirical estimates were quite similar in all cases.

Although applicable to a wide range of observational studies our approach has four major limitations. The first is a very practical one: our calculations require an estimate of the correlation $r_{Y_{1}Y_{2}}$ (or, alternatively, the covariance) between the baseline and the follow-up values, a number which is rarely given in papers. Imputing a plausible fixed value for $r_{Y_{1}Y_{2}}$ does not seem to be an adequate solution as the results extremely depend on its exact value as can be seen in example 3. Consequently, for most studies the original individual data for each person is needed.

Second, the interpretation of the graph p(*μ*) is limited as the reported p-values are not adjusted for multiple testing. Thus, the technique proposed is a exploratory data analytic strategy and should not be taken as proof of a treatment effect.

Third, in practical situations it might happen that $r_{Y_{1}Y_{2}}$, the estimator of *ρ*, is larger than 1. Indeed, in example 1 we found $r_{Y_{1}Y_{2}}$ = 1.111 for all *μ *whch is an indicator that the model was misspecified and that some subgroups of the whole population gain more from the treatment than others (those with average baseline values). Mee and Chua [12] already pointed that this leads to an overestimation of the treatment effect for each fixed *μ*. Consequently, the respective test is anticonservative. As a result p(*μ*) will fall too often below the predefined level of significance and the region of *μ*'s showing a significant treatment effect will be too broad. For a more detailled discussion on how misclassification affects the modified t-test see [12].

**Figure 3:**
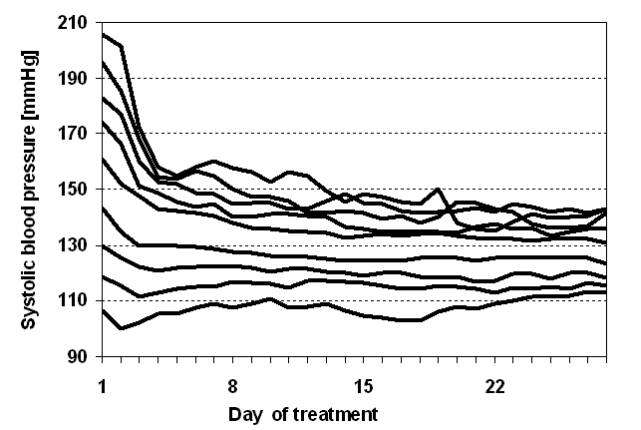
Redrawing of the blood pressure curves of Gutenbrunner and Ruppel [31].

Fourth, our approach is restricted to treatment effects which work additive on the mean. In contrast to this assumption, several complementary and alternative therapies are based on the therapeutic principle of "functional normalisation", i.e. they claim to actively exploit the self regulative capacities of the organism. In this sense, these approaches are assumed to have the potential not to shift a mean but to decrease high values and to increase low values to "normal" values, e.g. of blood pressure [31] or cardio-respiratory coordination [32]. This corresponds to a multiplicatively working treatment effect, a model first proposed by James [33] and extensively discussed by Senn and Brown [26,34], Chen and Cox [35], and Naranjo and McKean [36]. Again, it is difficult to distinguish such a treatment effect from RTM especially when data is collected selectively, for examples from the tails of a given distribution. This dilemma is quite illustrative in the example of Gutenbruner and Ruppel [31], redrawn in Fig. 3.

Here, the authors attribute the observed changes to an active process of the organism. However, building subgroups is a selection process by itself [37]. Thus RTM is likely to be present in this example. Consequently, one has to be aware, that also in situations where functional normalisation is assumed, RTM cannot be ignored. Our own simulation studies showed, that there is a high probability of erratiously deciding for normalisation when extreme values are more likely to be sampled. For example, if the correlation coefficient for repeated measurements is taken as 0.7 this error probability increases from more than 10% for a sample size of n = 20 to 55% for a sample size of n = 100 [38].

A multiplicative model of treatment effects also might help to solve Rogosa's problem when he considered populations which are not in a steady-state (see above). As the presence of a multiplicative factor alters the (unconditional) variance [26], unsteadiness can be interpreted as a treatment effect which pushes the second measurement values proportionally closer (or farther) to the mean according to the distance of first measurement values.

What we found to be evident from a broad variety of research papers is that the discussion of RTM affects all fields of life and behavioral sciences. Thus we were quite surprised, that methods to adjust for RTM are not very popular in medical data analysis. This is even more afflicting, if it is taken into account that especially in complementary medicine the discussion on appropriateness of study designs is quite vital. We would therefore like to encourage researchers to use methods like the one presented here (additional file 2) for the evaluation of uncontrolled studies to raise their methodological quality.

## Competing interests

The authors declare that they have no competing interests.

## Authors' contributions

TO wrote the initial draft of the manuscript. TO and RL calculated all mathematical elaborations. RL was responsible for all statistical algorithms and analyses of the examples. SNW was the guarantor of the project and interpreted the data from a medical point of view. All authors contributed to interpretation of the data and the critical revision of the manuscript, read and approved the final manuscript.

## Pre-publication history

The pre-publication history for this paper can be accessed here:



## Supplementary Material

Additional file 1 **SAS-Macro for the extended Mee-Chua t-test**. This macro is written in SAS code and calculates all statistics given in our paper based on individual raw data in a repeated measurement situation and also gives a graphical display of the test statistics. It was developed and tested under SAS version 9.1, although we believe it should give valid results in earlier releases. To run this macro it is necessary to have subscribed to the SAS modules BASE, STAT and SQL. Details how to run the macro can be found when opening the program code in an appropriate text editor. Click here for file

Additional file 2 **MS-EXCEL sheet for the extended Mee-Chua t-test**. This is an EXCEL 2000 sheet which calculates all statistics given in our paper based on means, standard deviations, and correlations in repeated measurement situations. Moreover, it provides a graphical display of the test statistics. Click here for file
